# Inoculation of grape musts with single strains of *Saccharomyces cerevisiae* yeast reduces the diversity of chemical profiles of wines

**DOI:** 10.1371/journal.pone.0254919

**Published:** 2021-07-22

**Authors:** Christian Philipp, Bahareh Bagheri, Micha Horacek, Phillip Eder, Florian Franz Bauer, Mathabatha Evodia Setati

**Affiliations:** 1 Department of Chemistry and Quality Control, Höhere Bundeslehranstalt und Bundesamt für Wein- und Obstbau, Klosterneuburg, Austria; 2 Department of Viticulture and Oenology, South African Grape and Wine Research Institute, Stellenbosch University, South Africa; 3 Department of Lithospheric Research, Vienna University, Vienna, Austria; University of Torino, ITALY

## Abstract

Anecdotal evidence suggests that spontaneous alcoholic fermentation of grape juice is becoming a more popular option in global wine production. Wines produced from the same grape juice by inoculation or spontaneous fermentation usually present distinct chemical and sensorial profiles. Inoculation has been associated with more similar end-products, a loss of typicity, and lower aroma complexity, and it has been suggested that this may be linked to suppression of the local or regional wine microbial ecosystems responsible for spontaneous fermentations. However, whether inoculated fermentations of different juices from different regions really end up with a narrower, less diverse chemical profile than those of spontaneously fermented juices has never been properly investigated. To address this question, we used grape juice from three different varieties, Grüner Veltliner (white), Zweigelt (red), and Pinot noir (red), originating from different regions in Austria to compare spontaneous and single active dry yeast strains inoculated fermentations of the same grape samples. The chemical analysis covered primary metabolites such as glycerol, ethanol and organic acids, and volatile secondary metabolites, including more than 40 major and minor esters, as well as higher alcohols and volatile fatty acids, allowing an in depth statistical evaluation of differences between fermentation strategies. The fungal (mainly yeast) communities throughout fermentations were monitored using automated ribosomal intergenic spacer analysis. The data provide evidence that inoculation with single active dry yeast strains limits the diversity of the chemical fingerprints. The fungal community profiles clearly show that inoculation had an effect on fermentation dynamics and resulted in chemically less diverse wines.

## 1 Introduction

The transformation of grape must to wine occurs by alcoholic fermentation in which the grape sugars, glucose, and fructose are converted by yeasts, theoretically, into 51.1% ethanol and 48.9% carbon dioxide [1, 2]. Yeasts are not only responsible for the metabolism of sugar to alcohol and CO_2_, but they also play an equally important role in the formation of secondary metabolites and in the conversion of aroma precursors into volatile aromas [3–8]. Alcoholic fermentation traditionally occurs spontaneously and it is initiated by a diverse community of indigenous yeasts of the genera *Hanseniaspora*, *Pichia*, *Metschnikowia*, *Candida*, *Torulaspora*, *Rhodotorula*, *Cryptococcus*, *Lachancea*, *Zygosaccharomyces* and most importantly, *Saccharomyces* [9]. The spontaneous fermentation process is often characterized by successional development of yeast genera, species and strains, with the final stages of fermentation usually dominated by strains of *Saccharomyces cerevisiae*. Since the diverse communities in spontaneous fermentations express different phenotypic traits, it is generally assumed [7] that the wines they produce are consequently organoleptically more diverse and perhaps more complex. However, spontaneous fermentation processes are also regarded as inconsistent, may tend to get stuck or sluggish and have a higher risk of off-flavours [10]. Consequently, since the 1960s, alcoholic fermentation on an industrial scale is typically conducted through inoculation with commercial Active Dry Yeast (ADY) strains [11–14] that have high fermentation capacity and proven capability to produce wines with consistent quality and predictable aroma compositions. The large inoculum of active *S*. *cerevisiae* cells in most cases ensures a very rapid dominance of a single strain, and will therefore likely reduce any impact the natural microbiota may have had if allowed to ferment spontaneously. The use of starter cultures is therefore thought to reduce aroma diversity in wines originating from different regions [10, 15–17]. However, to the best of our knowledge, no studies have provided direct evidence for such reduction in wine flavour diversity.

Studies have evaluated the effect of grape regional-specific microbiome on chemical and aromatic properties of wine. The effect of grape cultivar and geographical location in shaping the grape microbiome have already been reported [18, 19]. The current study aimed to compare the chemical fingerprint of wines produced by spontaneous and inoculated fermentation of several juices derived from 3 prominent Austrian grape varietals (Grüner Veltliner, Zweigelt, and Pinot noir) and two important Austrian wine regions (Lower Austria and Burgenland) to determine whether the chemical fingerprints are more similar to each other in inoculated wines than in spontaneous fermentation. For this purpose, fungal and yeast biodiversity profiles were evaluated at different stages of fermentations, and the final products were chemically characterised. The chemical analysis covers primary metabolites including glycerol, ethanol and organic acids, and biogenic amines, and a large number of volatile secondary metabolites, including more than 40 major and minor esters, as well as higher alcohols and volatile acids. This allows us to perform an in depth statistical evaluation of differences between fermentation strategies that have not been reported in previous studies.

## 2 Materials and methods

### 2.1 Vineyard locations and grape material

Three different grape varieties were obtained from two regions in Austria. Grüner Veltliner (GV) K and Pinot noir (PN) were obtained from the Federal College and Research Center in Klosterneuburg (Klosterneuburg, Lower Austria, acronym K) and the Grüner Veltliner B and Zweigelt (ZW) from the winery Scheiblhofer (Andau, Burgenland, acronym B). The experiment was carried out with grapes from the 2017 vintage. All vineyards were cultivated according to the requirements of Integrated Production according to Austria’s Agri-environmental program (ÖPUL). Since it is a matter of comparing fermentations and not necessarily of local differences, it is not mandatory to coordinate the pesticide applications.

### 2.2 Grape processing and vinification

A total of 350 kg of grapes per varietal was delivered in a large box from each location. The grapes were destemmed and crushed with a destemmer (Fuhrmann, Steinebrunn, Austria) and during this process 50 mg/kg SO_2_ was added. Sulphurization mainly kills off many of the bacterial species and thus reduces the risk of spoilage. The impact on the yeast community is more limited, and only some non*-Saccharomyces* species are primarily affected [11]. After the destemming, the white grapes were pressed at a maximum pressure of 1.5 bar using a TPG 500 wine press (Wottle Anton Maschinen–u. Weinpressenbau GmbH, Poysdorf, Austria). The machines, boxes and containers were sprayed before harvest and in between and before each batch with an alkaline and hydrogen peroxide-reinforced foam cleaner (Foam Caustic and Bisteril, each 5 percent, Manufacturer: Thonhauser, Perchtoldsdorf, Austria). Each of the juices was mixed with 2 g/hL pectolytic enzyme (Lallzym HC, Lallemand, Blagnac, France) for clarification and placed in a 10°C refrigeration room overnight, following which the clarified juice was siphoned from the lees into another tank for homogenization and then divided into six glass balloons, each with a capacity of 34 L. Before usage, the glass containers were cleaned by filling with ahot two-percent acidic SO_2_ solution for at least 12 hours to minimize contamination from fermentation vessels. For the red grape samples, after destemming and crushing, the mash of each sample was homogenized in a large crate and divided into 6 plastic fermentation containers wherein fermentation was performed. The plastic containers were treated as glass balloons before usage to minimize microbial contamination.

For each grape sample, the 6 fermentation vessels containing the sample were divided into two sets of fermentations, inoculated and spontaneous, and thus the experiment performed in triplicate. For the inoculations, *Saccharomyces cerevisiae* (*var bayanus*) ADY strain, Oenoferm® Freddo (Ersblöh, Geisenheim, Germany) was used for red and white wines to address our goal. Red wines were fermented in a controlled manner at 25°C, whereas white wines were fermented at 18°C. The fermentations were considered complete when the relative density remained unchanged over three consecutive days, and they were considered to have fermented dry when the residual sugar was below 6 g/L as defined by the Austrian Wine Law [20].

The fully fermented red wines were pressed with a hydro-press with a maximum pressure of 2.85 bar. The white and red wines were transferred into storage containers and sulphur dioxide was added to a final concentration of 50 mg/L free SO_2_. After two weeks of stabilization, the wines were subjected to sterilisation filtration using candle filters, bottled and stored at 15°C until analysis.

### 2.3 Chemical analyses

#### 2.3.1 Basic parameters

Fourier-transform infrared spectroscopy (FTIR) was used according to OIV/OENO Resolution 390/2010 (International Organization of Vine and Wine, 2010) using FOSS WineScan (FT 120 Reference Manual, Foss, Hamburg, Germany) to determine sugar, total acid, pH and yeast assimilable nitrogen (YAN) in the grape must samples. Similarly, relative density, available alcohol (vol %), sugar (sum of glucose and fructose, g/L), titratable acids (g/L), tartaric acid (g/L), and lactic acid (g/L) in the finished wines were measured using FOSS wineScan. Overall, the musts did not show significantly different sugar levels, but YAN content varied considerably (Table 1). The Grüner Veltliner K had insufficient YAN (YAN <150 mg/L) (Dittrich and Großmann, 2010). However, the addition of organic or inorganic nitrogen was deliberately avoided, so that any difference in the degree of fermentation of the varieties (pure yeast vs. spontaneous fermentation) could be determined. The fermentation progress and the various phases of fermentation were monitored using a flexural resonator (DMA 35, ANTON PAAR, AUSTRIA).

**Table 1:** Standard wine chemical parameters of the grape juices: Different letters in the same column mean significant differences between the variants.

Cultivar	Vineyard	°Brix	pH	Total acid (g/L)	YAN (mg/L)
Grüner Veltliner K	Lower Austria	20,59^a^±0,00	3.28^bc^±0,00	4.67^a^±0,03	155^b^*^1^±2
Grüner Veltliner B	Burgenland	21,76^a^±0,00	3.16^a^±0,01	6.60^b^±0,00	113^a^*^1^±3
Zweigelt	Burgenland	20,56^a^±0,00	3.38^c^±0,00	5.89^a^±0,00	239^d^*^1^±3
Pinot noir	Lower Austria	20,59^a^±0,06	3.19^ab^±0,01	5,53^ab^±0,03	206^c^*^1^±8

*1 using TukeyB test, the rest using Kruskal-Wallis test and pairwise comparisons.

The glycerol content of wines was determined using an automated enzymatic analysis (Konelab 29, Thermo Fisher, Waltham, USA) as described by Blouin and Dubernet [21] whereas the volatile acids content was determined according to the type 1 method of OIV, OIV-MA-AS313-02 [22] using a semi-automatic distillation apparatus (digital distilling unit Super Dee, Gibertini Elettronica S.R.L. Milan, Italy) and a Lieb Zacherl glass apparatus (Neubert Glas, Geschwenda, Germany).

#### 2.3.2 Organic acids

The contents of the most common acids (tartaric acid, malic acid, lactic acid, volatile acid, succinic acid, citric acid) in wines were determined by ion chromatography. The separation was performed using a Dionex ion-exchange chromatography (AS 50 Autosampler, GP 40 Gradient Pump, CD 20 Conductivity Detector; DS 3 Detection Stabilizer; Thermo Fisher, Waltham, USA). ATC-3 as a precolumn, AG 11 (4 mm) AS 11 (4 mm) as a separation column and AMMS III 4 as a suppressor was used. As a regeneration solution, 12.7 mM H_2_SO_4_ was used. The cycle was made with a peristaltic pump (flow at 3–4 mL/min). The samples were diluted according to the concentration of malic acid, tartaric acid, or citric acid (usually 1:100). They were filtered through an RP-18 tube to remove interfering phenols and dyes (RP-18 tube: the first filtration of the day was activated with HCl / CH_3_OH mixture (1 mL HCl / 100 ml CH_3_OH) and rinsed with water. The eluents used (A: 0.2 mM NaOH, B: 5.0 mM NaOH, C: 38.0 mM NaOH, D: CH_3_OH) were degassed with helium for 15 min). Calibration with external standards was done according to good laboratory practice. Chemical standards were procured from Sigma-Aldrich (St. Louis, USA). The correlation coefficients of the calibration were between 0.996 and 0.999. The limit of quantification (LOQ) varied between 0.17 mg/L and 2.25 mg/L in diluted samples depending on the acid, standard error by 10 repeats of the same samples was less than 10% for each component.

The total acid calculated as tartaric acid was analysed according to OIV / OENO Resolution 390/2010 using FOSS-WineScan (FT 120 Reference Manual, Foss, Hamburg, Germany) [23].

#### 2.3.3 Volatile organic compounds analysis

For the analysis of the different aromatic substances, two gas chromatographs from Agilent Technologies (Santa Clara, USA) were used. The first system consisting of a 6890 N GC system with a 5975 inert mass selective detector and a CTC Analytics Autosampler (Zwingen, Switzerland) was equipped with a ZB-Wax Plus column (length: 60 min, ID: 0.25 mm, df = 0.25 μm) from Phenomenex (Torrance, USA) and was used to analyse the higher alcohols, volatile carbonic acids and ethyl acetate, ethyl lactate and diethyl succinate [24]. The second system consisting of a 7890A GC system with a 5975C inert MSD with a triple axis detector and a CTC Analytics autosampler (Zwingen, Switzerland), was used to analyse major and minor ester compounds. This system was equipped with a ZB-5MS column (length: 60 min, ID: 0.25 mm, df = 0.25 μm) from Phenomenex (Torrance, USA). Minor and major esters in the wine samples were analysed using stable isotope dilution assay headspace solid-phase microextraction gas chromatography-mass spectroscopy (SIDA-HS-SPME-GC-MS) [25, 26]. All analyses were done in duplicate from two bottles. Quantification was done using the relative ratio of the peak area of the analytical standard to the peak area of the internal standard. Of all compounds, analytical grade substances were available. Chemical standards and internal standards were procured from Altmann Analytics-Shop (Fluka products, Munich, Germany), Sigma-Aldrich (St. Louis, USA), Merck Schuchardt (Hohenbrunn, Germany), Moellhausen (Vimercate, Italy), Roth (Karlsruhe, Germany) and Honeywell Specialty Chemicals Seelze GmbH (formerly Riedel-de Haën, Seelze, Germany) and showed all the maximum available concentration (90%–99.5%). All non commercially available standards were produced by on-site synthesis [27, 28]. The validated data for both methods are shown in S1 Table. Calibration was done in synthetic wine, validation was done in a commercial Pinot blanc wine of vintage 2017, repeatability using the standard error of 10 repeats of the same sample and LOQ and LOD through signal/noise relation.

#### 2.3.4 Fungal community fingerprinting

The samples for the fungal community fingerprinting analyses were taken at the start of fermentation (SF), during mid-alcoholic fermentation (MF) and at the end of alcoholic fermentation (EF). The progress of the fermentation and the different phases of the fermentation were monitored with a bending oscillator. Fermentation started at the first noticeable reduction in density (SF). Middle of fermentation (MF) was after a significant reduction of more than 50% from the initial value and end of fermentation (EF) was when the density value had not changed for three days. For the community analysis, fifty milliliters of must (respectively grape juice before and wine after fermentation) was centrifuged at 6000 *g* for 5 min, followed by re-suspension of the solid matter containing microbial biomass in 5 mL of wash solution containing 0.15 M NaCl, 0.1 M EDTA and 2% (w/v) Polyvinyl pyrrolidone [29]. Genomic DNA was extracted from 200 μL of the cell suspension using the FavorPrep™ soil DNA Isolation Mini Kit (Favorgen, Biotech Corp) according to the manufacturer’s instructions with minor modifications. For instance, the samples were vortexed for 3 min with glass beads for lysis instead of the recommended 1 min and the DNA was eluted with 25 μL. The DNA was quantified spectrophotometrically, using the NanoDrop® ND-1000 (NanoDrop Technologies Inc., Wilmington, DE, United States). DNA integrity was assessed by gel electrophoresis on 1% (w/v) agarose gel with 1 x TAE (Tris-Acetic acid-EDTA) buffer as the mobile phase and visualised under UV with Gel Red staining. The ITS1-5.8S rRNA-ITS2 gene region was amplified from 50 ng DNA template by Polymerase Chain Reaction (PCR) using carboxy-fluorescein labelled ITS1 (5′-[FAM] TCCGTAGGTGAACCTGCGG-3′) and ITS4 (5’-TCCTCCGCTTATTGATATGC-3’) primer [30]. The PCR amplicons were mixed with the GeneScan™ 1200 LIZ® Size Standard (Thermo Fisher Scientific) and separated by capillary electrophoresis using the Applied Biosystems 3130×l Genetic Analyzer (Applied Biosystems, Forster City, CA, USA). The data were pre-processed with Gene Mapper 4.0 software (Applied Biosystems, Forster City, CA, USA) and further analysed on the T-RFLP analysis Expedited (T-REX) online software [31]. Peaks were detected from 50 bp to 1000 bp based on the molecular standard. However, only the peaks ranging between 300 to 900 bp, with a fluorescence intensity greater than 50 units and peaks represented more than 0.5% of the total fluorescence were considered for further analysis. ARISA was performed on triplicate samples and a peak was considered present and analysed as an Operational Taxonomic Unit (OTU) if it appeared in at least two of the replicates. The ARISA fingerprinting profiles were standardized by dividing each peak area by the total area of the peaks in a sample profile. The generated relative abundance data was further explored by Principal Component Analysis (PCA) performed on the variance-covariance matrix.

### 2.4 Statistics

All experiments were performed in triplicates including 3 biological and 2 technical repeats. The statistical evaluation of the data was carried out using SPSS-Statistics 22.0 (IBM). First, the data set was tested for normal distribution using an exploratory data analysis. If there were a normal distribution and variance homogeneity of the data, ANOVA tested for significant differences (α<0.05) between the two fermentation variants in the respective variety group and origin. If a condition was violated, non-parametric testing according to Mann Withney U was used for testing for significant differences (α<0.05). These tests did not attempt to identify significant differences between the varieties since the results of the chemical analysis parameters were further subjected to a categorical principal component analysis (PCA) and presented as a biplot. For this purpose, the method of object principal normalization was chosen and a grouping of a maximum of seven was set and a 95% confidential ellipse was estimated by boot stream based on the given data. The two dimensions of the biplot representation are given with the variance coverage [32].

## 3 Results

### 3.1 Fermentation process and degree of fermentation

The fermentations were continuously monitored by measuring the relative density. The fermentation dynamics can be seen in S1 Fig. Overall, all inoculated juices fermented dry (lower 6 g/L residual sugar) and the inoculated Grüner Veltliner B and Zweigelt fermented significantly faster than the spontaneously fermented equivalents while the fermentation curves of the spontaneous fermentations for all variants show a delayed alcoholic fermentation at the beginning and in the middle section. Among the spontaneous fermentations, the Grüner Veltliner B that had low YAN levels in the must become stuck and fermentations were terminated with ≈ 60 and 29 g/L residual sugar, respectively, and thus contained significantly lower ethanol levels compared to their inoculated variants. The Zweigelt and Pinot Noir contained considerably higher glycerol than the Grüner Veltliner wines. Similar to the ethanol trends, the Grüner Veltliner B displayed significantly lower concentrations of glycerol in the spontaneously fermented wines compared to the inoculated ones (data not shown).

### 3.2 Fungal population dynamics

**Fig 1:**
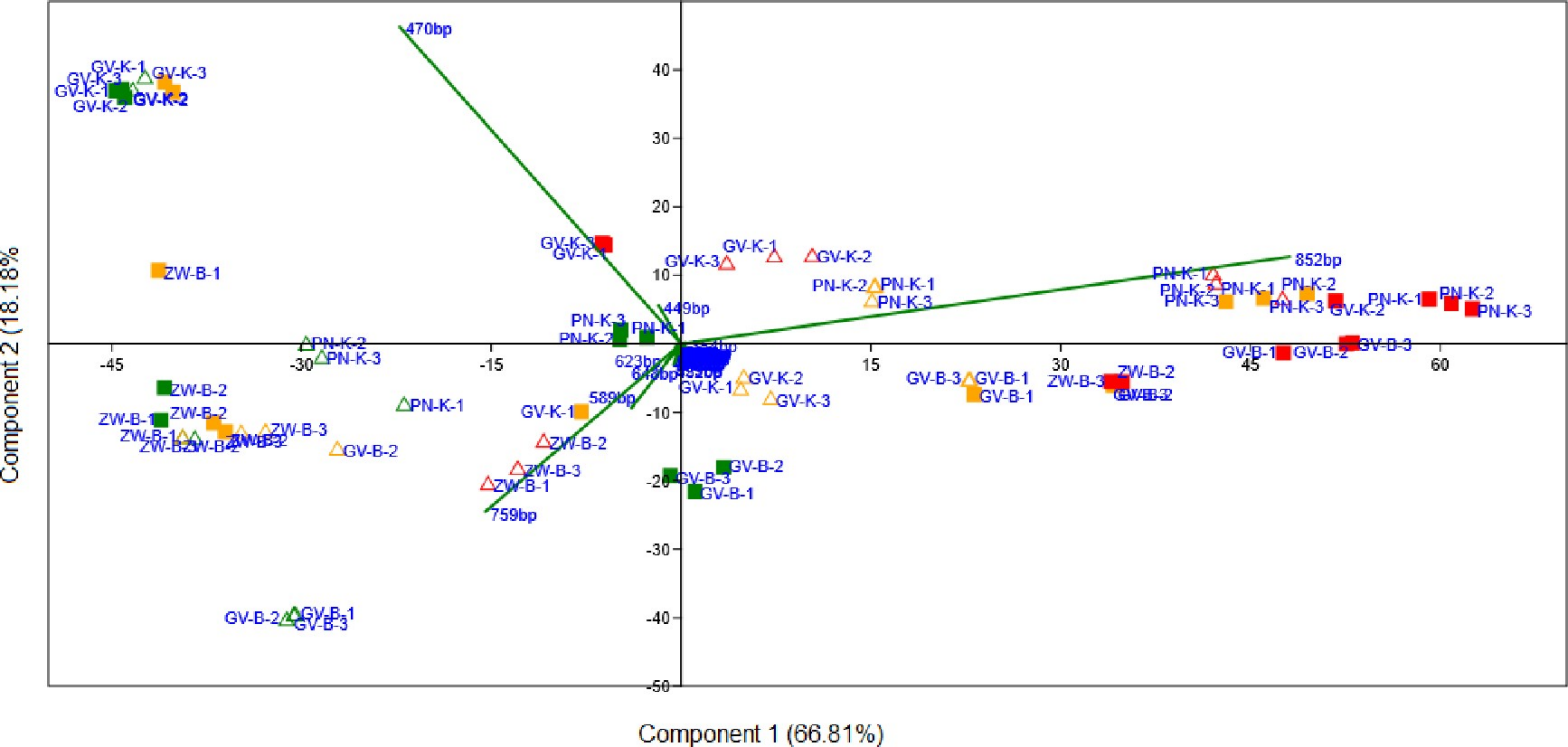
PCA biplot comparing the population dynamics. The start (green), middle (orange) and end of fermentation (red), inoculated (filled square) spontaneous (triangle) variants, Zweigelt from Burgenland (ZW-B), Grüner Veltliner from Burgenland and Klosterneuburg (GV-B and GV-K), Pinot not from Klosterneuburg (PN-K). Three replicates were analysed for each fermentation and represented numerically (1,2,3).

A PCA biplot was used to visualize the change in the fungal community patterns throughout fermentation (Fig 1). PC1 which explained 66.81% of the variance allowed for separation of the samples according to fermentation stage, while PC2 which explained 18.18% of the variance, separated samples based on the origin of the wines. Interestingly, the different fermentation strategies of these wines did not influence the overall pattern significantly, while the two variants of Zweigelt wines differed strongly only at the end of fermentation (EF), the other cultivars already showed differences in earlier stages. Samples from Burgenland and Klosterneuburg form separate clusters. Regarding the loadings, OTU 852 bp was the main driver of the separation according to the fermentation stage, while OTU 759 bp (Burgenland) and 470 bp (Klosterneuburg) were mainly responsible for the separation of the samples based on the origin (Fig 1).

**Fig 2:**
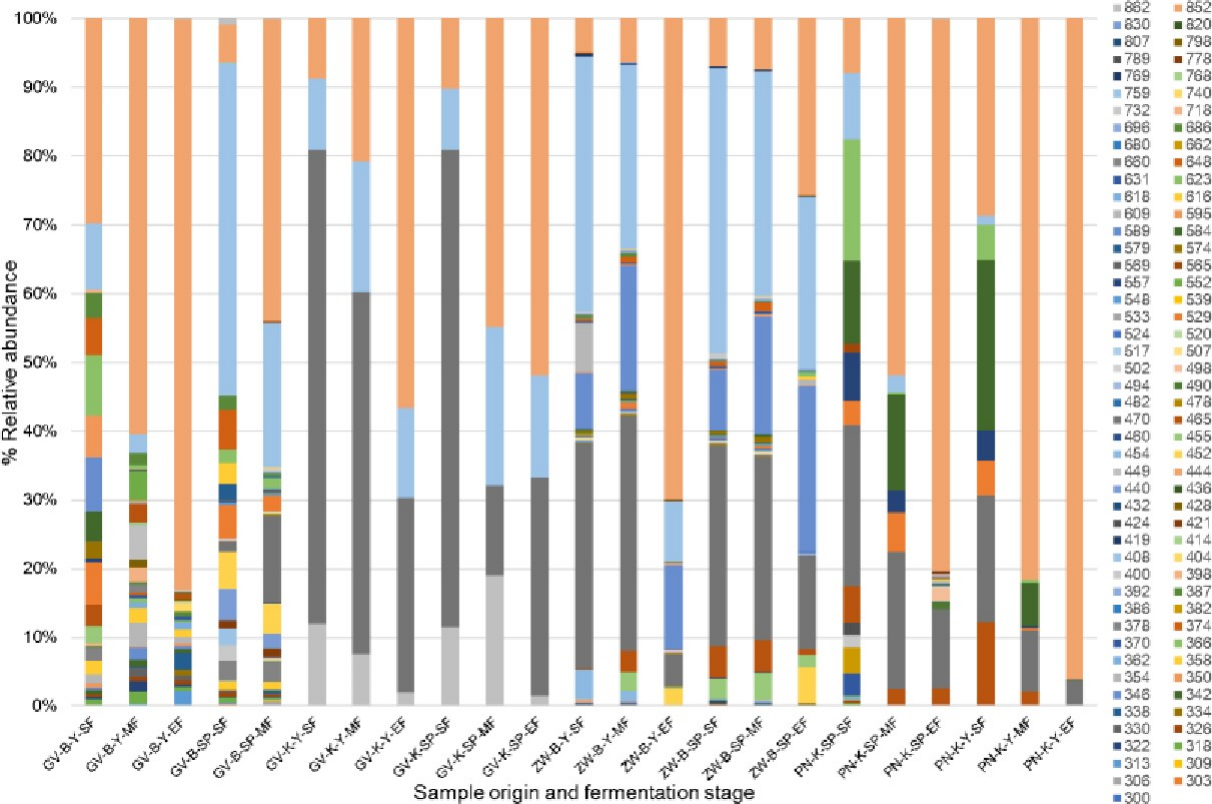
Population dynamics showing fungal OTU development. Start (SF), middle (MF) and end (EF) of fermentation. Zweigelt from Burgenland (ZW-B), Grüner Veltliner from Burgenland and Klosterneuburg (GV-B and GV-K), Pinot not from Klosterneuburg (PN-K). the samples GV-B-SP-EF are missing because the fermentation has stuck.

OTU 470 bp was detectable at the start of fermentation in all samples except the Grüner Veltliner from Burgenland. This OTU was the most abundant in both inoculated and spontaneous fermentation samples of the Grüner Veltliner from Klosterneuburg, accounting for more than 60% of the population and remained persistent till EF, accounting for approximately 30% of the population in both variants. OTU 759 bp was detected in all SF samples but was most dominant in the spontaneous variant of the Grüner Veltliner from Burgenland where it accounted for 48% of the population and remained persistent until MF at high abundance. This OTU was also dominant at SF in the Zweigelt samples where it accounted for 37% and 41% of the population in the inoculated and spontaneous variants, respectively and remained at levels above 20% of the population at MF in both variants and persisted at these levels until EF in the spontaneous variant (Fig 2).

**Fig 3:**
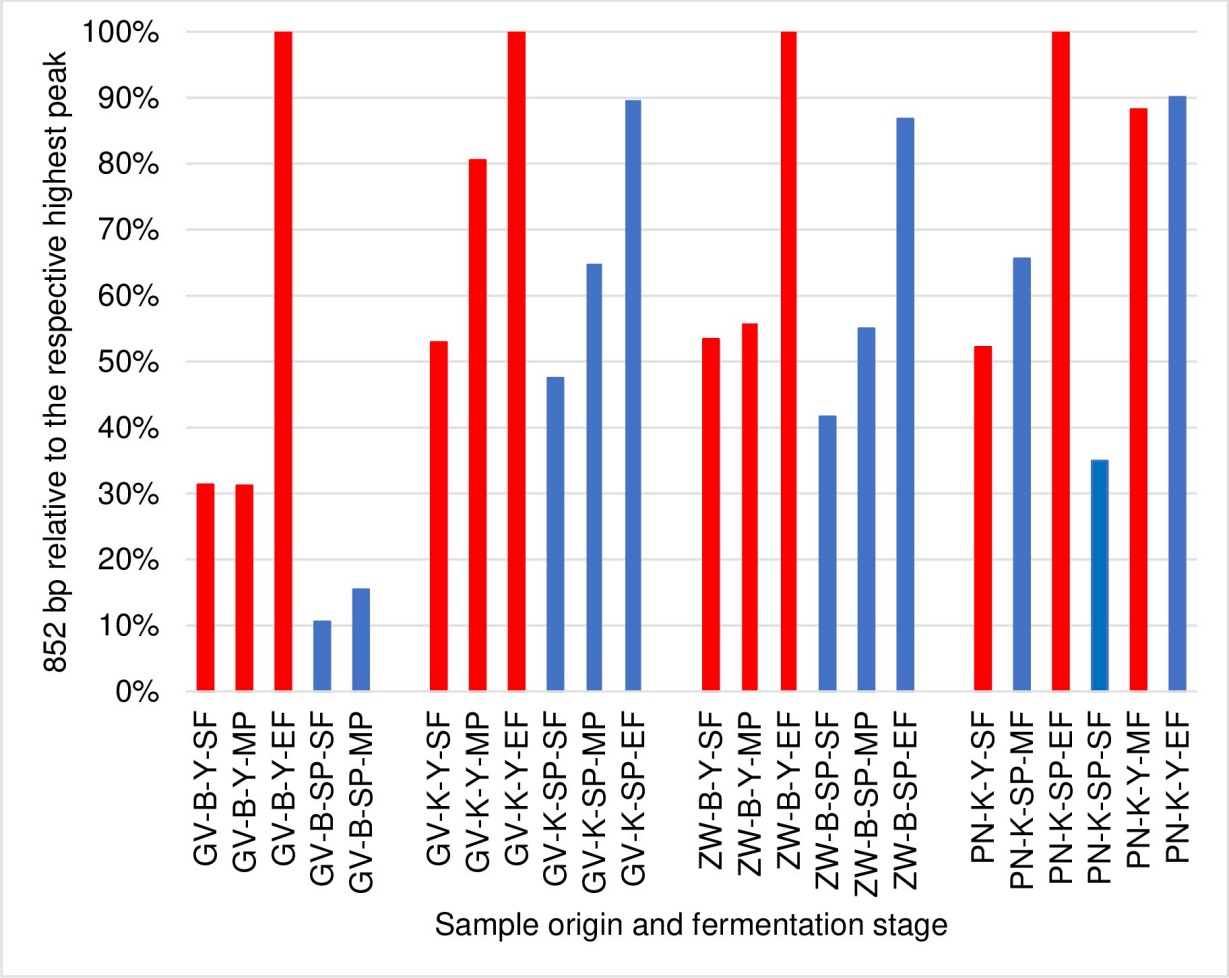
Population dynamics: Showing relativ fungal OTU 852 development. In releation to the respective highest peak from the start (SF), middle (MF) and end (EF) of fermentation: red means a higher peak compared to the corresponding variant in the same fermentation stage; the samples GV-B-SP-EF are missing because the fermentation has stuck.

OTU 852 bp showed good implantation in the inoculated Pinot noir and Burgenland Grüner Veltliner variant accounting for nearly 30% of the population at SF and rapidly became the dominant OTU accounting for 60% in the Grüner Veltliner and 82% in the Pinot noir by MF, and for 83% and 96% at EF, respectively. Considering the ARISA peak areas of the OTU 852 bp compared to the highest peak of all measurements for one grape variety (Fig 3), it is obvious that this yeast strain establishes more rapidly and was significantly more dominant in the inoculated grape musts with the single exception of the PN SP MF sample. These data confirm that the yeast microbiota differs significantly among the two fermentation strategies and that the inoculation affected the fermentation dynamics. This statement is further confirmed by the different fermentation curve of the innoculated variants compared to the spontaneously fermented variants (S1 Fig).

### 3.3 Non-volatile fruit and metabolism acids

Table 2 shows the average contents and standard deviations of the spontaneously fermented and the inoculated wines. Our data showed no clear differences in the content of tartaric acid and citric acid between spontaneously fermented and inoculated wines, but the succinic acid, malic acid, and the titratable total acid content were significantly higher in the inoculated fermentation wines. In Grüner Veltliner B and Pinot noir, the spontaneously fermented wines showed significantly higher levels of lactic acid. Nevertheless, complete malolactic acid degradation did not occur in any variant.

**Table 2:** Content of acids and higher alcohols in the wines.

Parameters	GVBY (n = 3)	GVBSP (n = 3)	GVKY(n = 3)	GVKSP(n = 3)	ZWY(n = 3)	ZWSP(n = 3)	PNY(n = 3)	PNSP(n = 3)
**Acids (g/L)**								
total acid	6.03^b^±0.06	5.83^a^±0.12	4.53^b^±0.06	4.27^a^±0.06*^1^	5.23^a^±0.15	5.20^a^±0.17	4.73^a^±0.06	4.71^a^±0.06*^1^
tartaric acid	2.67^a^±0.06	2.93^a^±0.12	2.40^a^±0.00	2.40^a^±0.00	1.70^a^±0.10	1.60^a^±0.10	0.73^a^±0.06	0.83^a^±0.06
malic acid	1.53^b^±0.06*^1^	1.20^a^±0.00*^1^	0.73^b^±0.06	0.60^a^±0.00	1.17^a^±0.15	0.10^a^±0.10	1.37^a^±0.06	1.27^a^±0.06
lactic acid	nd^a*1^	0.23^b^±0.06*^1^	nd	nd	0.27^a^±0.12	0.33^a^±0.06	0.13^a^±0.06	0.33^b^±0.06*^1^
citric acid	0.12^a^±0.00	0.12^a^±0.00	0.13^a^±0.00	0.13^a^±0.01	0.23^a^±0.00	0.21^a^±0.01	0.37^a^±0.00	0.38^a^±0.01
succinic acid	0.77^b^±0.02	0.68^a^±0.01	0.85^b^±0.03	0.65^a^±0.05	1.13^b^±0.03	0.96^a^±0.07	0.94^a^±0.04	0.90^a^±0.02
volatile acids by means of titration	0.50^a^±0.00	0.53^a^±0.06	0.27^a^±0.06	0.29^a^±0.06	0.20^a^±0.17	0.37^a^±0.15	0.37^a^±0.06	0.50^b^±0.00
**Minor volatile acids (mg/L)**								
Butyric acid	0.63^a^±0.07	1.24^b^±0.27	1.29^a^±0.13	1.52^a^±0.10	0.44^a^±0.02	0.36^a^±0.06	0.51^a^±0.13	0.54^a^±0.12
Isobutyric acid	2.76^b^±0.46	1.98^a^±0.48	2.53^b^±0.30	1.38^a^±0.23	3.07^a^±0.32	2.31^a^±0.58	2.18^a^±0.52	1.67^a^±0.61
Isovaleric acid	0.90^b^±0.28	0.25^a^±0.08	0.48^b^±0.11*^1^	0.27^a^±0.04*^1^	0.31^a^±0.08	0.31^a^±0.14	0.38^a^±0.10	0.38 ^a^ ±0.12
Hexanoic acid	1.12^a^±0.33	2.47^b^±0.16	2.19^a^±0.07	4.39^b^±0.38	0.85^b^±0.02	0.68^a^±0.08	0.88^a^±0.17	1.56^b^±0.10*^1^
Octanoic acid	1.07^a^±0.51	0.93^a^±0.27	2.16^a^±0.54	8.30^b^±1.81	0.54^a^±0.18	0.59^a^±0.11	0.76^a^±0.19	1.53^b^±0.26
Decanoic acid	1.89^b^±0.58	0.47^a^±0.15	1.62^a^±0.38	3.12^a^±1.08	0.28^b^±0.04	0.15^a^±0.06	0.84^b^±0.32	0.39^a^±0.22
**Higher alcohols (mg/L)**								
1-propanol	35.82^b^ ±3.61	23.19^a^±1.99	27.01^b^ ±1.58	16.68^a^±0.20	118.99^a^±13.17	99.60^a^±23.00	35.12^a^±2.73	76.38^b^±4.25
Isobutanol	18.94^a^±1.50	19.69^a^±2.51	25.55^a^±1.26	29.05^b^±1.22	73.77^a^±13.05	82.24^a^±15.48	40.65^a^±1.68	78.25^b^±3.83
Isoamyl alcohol	91.70^a^±11.48	83.54^a^±11.27	171.99^a^±20.73	159.96^a^±14.66	146.08^a^±25.25	121.20^a^±20.86	232.89^a^±18.40	254.59^a^±27.17
1-butanol	0.30^b^±0.03	0.20^a^±0.02	0.48^b^±0.03	0.43^a^±0.04	0.85^b^±0.06	0.73^a^±0.04	1.16^a^±0.07	1.07^a^±0.04
1-hexanol	0.80^a^±0.10	0.91^a^±0.08	2.16^a^±0.42	2.14^a^±0.08	1.31^a^±0.05	1.37^a^±0.09	1.63^a^±0.24	1.78^a^±0.18

Parameters are given with their mean and standard deviation. For each volatile substance different letters in the same variant (grape and origin) indicate significant differences between variants. (ANOVA p≤0.05; *^1^ Mann Withney U-Test p≤0.100); nd = not detectable.

### 3.4 Volatile acidity and ethyl acetate

The volatile acidity, calculated as the concentration of acetic acid and ethyl acetate, serves as an indicator of spoilage [11]. In the current study, only Pinot noir wine displayed a significant difference in the content of acetic acid, with higher content in the spontaneously fermented wines than in inoculated wines (Table 2).

Regarding the content of ethyl acetate, the level was consistently higher in the spontaneously fermented wines, specifically in Grüner Veltliner B (Table 2).

### 3.5 Minor volatile acids

The content of carboxylic acids is shown in Table 2. The spontaneously fermented wines had significantly higher contents of hexanoic acid than the wines produced with ADY except for the Zweigelt, which showed significantly lower levels. In contrast, the inoculated wines had higher concentrations of decanoic acid compared to the spontaneously fermented wines. Regarding the short-chain acids and the carboxylic acids butyric acid, isobutyric acid, and isovaleric acid, which are often associated with negative aromas, no consistent trend was observed. With butyric acid, only the Grüner Veltliner B wines showed a significant difference with higher contents in the spontaneously fermented wines.

### 3.6 Higher alcohols

In the Pinot noir, a significantly higher average content of 1-propanol could be found in the spontaneously fermented wines, whereas the opposite pattern was observed in the Grüner Veltliner B and Zweigelt. The content of 1 hexanol also varied notably between the wines. The spontaneously fermented wines consistently showed a higher content of isobutanol than the inoculated wines. However, the differences in the content of isobutanol, isoamyl alcohol, 1 butanol and benzyl alcohol between the spontaneously fermented and the inoculated wines were smaller than the differences in the content of 1-propanol and 1-hexanol (Table 2).

### 3.7 Ester compounds

Forty three different ester compounds were detected in the course of this work (Table 3). Significant differences between the spontaneously fermented and inoculated wines were found for specific compounds in all ester groups. Twenty three compounds showed a significant difference in Grüner Veltliner B whereas twenty in Grüner Veltliner K and ten compounds in Pinot noir and Zweigelt displayed a significant difference.

**Table 3:** Content of ester compounds in the wines.

Parameters	GVBY (n = 3)	GVBSP (n = 3)	GVKY(n = 3)	GVKSP(n = 3)	ZWY(n = 3)	ZWSP(n = 3)	PNY(n = 3)	PNSP(n = 3)
**Major Ethyl esters (mg/L)**								
Ethyl acetate	132.23^a^±46.16	282.54^b^±82.01*1	67.98^a^±12.26	92.59^a^±25.88	218.34^a^±63.05	238.2^a^±41.22	118.33^a^±59.68	138.22^a^±44.60
Diethyl succinate	4.12^b^±1.20*^1^	2.94^a^±0.17*^1^	2.89^b^±0.62	1.92^a^±0.43	3.22^a^±0.70	3.13^a^±1.27	1.76^b^±0.71	1.50^a^±0.34
Ethyl lactate	69.61^a^±15.63	42.67^a^±10.83	14.42^a^±3.11	16.35^a^±2.77	66.07^a^±14.21	74.41^a^±23.51	34.52^a^±2.26	54.24^b^±8.93
**Minor Ethyl esters (μg/L)**								
Ethyl butyrate	122.73^b^±6.79	104.67^a^±5.50	114.32^a^±10.17	183.17^b^±14.29	115.68^b^±2.02	93.39^a^±3.83	92.27^a^±7.32	225.21^b^±18.80
Ethyl hexanaote	164.63^a^±6.50	175.76^a^±9.86	182.53^a^ ±17.61	312.07^b^±17.04	79.08^a^±13.39	58.95^a^±11.66	103.31^a^±7.39	140.46^b^±15.11
Ethyl octanoate	764.75^b^ ±42.41	499.61^a^±60.40	108.14^a^ ±14.15	222.06^b^±11.36	53.58^b^±18.66	27.64^a^±4.44	51.23^a^±8.78	42.77^a^±4.05
Ethyl decanoate	873.77^a^±160.49	1.071.94^a^±214.92	159.60^a^ ±22.70	251.03^b^ ±22.20	44.00^a^±8.32	36.90^a^±11.72	53.29^b^±10.70	21.45^a^±3.93
Ethyl laurate	16.39^a^±3.35	21.14^a^±4.15	12.63^a^±0.57	13.83^b^±0.77	12.99^a^±0.52	13.24^a^±2.68	10.64^a^±3.10	7.48^a^±0.78
Ethyl myristate	8.35^a^±2.08	16.00^b^±1.89	10.12^a^±1.00	13.12^b^±1.47	13.20^a^±0.88	15.70^b^±0.65	8.05^a^±2.58	6.60^a^±1.56
Ethyl palmiate	47.05^a^±7.48	67.47^b^±8.72	33.01^a^±3.39	52.45^b^±3.06	34.85^a^±1.84	43.01^b^±1.62	17.07^a^±4.81	15.21^a^±2.40
**Esters of branched aliphatic acids (μg/L)**								
Butyl isobutyrate	<0.98	<0.98	<0.98	<0.98	1.61^a^±0.05*^1^	1.63^a^±0.11*^1^	1.17±0.24	<0.98
Ethyl isovalerate	34.74^b^±1.10	11.69^a^±0.75	24.60^b^ ±1.45	9.25^a^±0.80	9.32^a^±2.52	6.09^a^±1.72	9.49^a^±0.55	9.28^a^±0.62
Propyl isovaleriate	<0.05	<0.05	<0.05	<0.05	<0.05	<0.05	<0.05	<0.10
**Higher alcohol acetate (μg/L)**								
Isobutyl acetate	<12.70	15.92±1.36	<12.70	26.92±3.29	33.51^a^±3.03	62.92^b^±3.32	<12.70	40.75±3.23
2-methylbutyl acetate	32.20^a^±1.97	30.22^a^±2.34	22.89^a^±1.88	62.89^b^±2.33	26.46^a^±4.23	27.61^a^±4.31	16.11^a^±1.92	39.12^b^±4.03
Isoamyl acetate	177.11^a^±7.82	237.31^b^±16.29	185.06^a^±17.70	628.09^b^ ±60.77	128.62^b^±1.06	109.68^a^±6.85	88.89^a^±14.63	278.82^b^±32.01
Pentyl acetate	<0.10	<0.10	<0.10	0.57 ±0.04	0.67^a^±0.05	0.60^a^±0.08	0.25^a^±0.04	0.33^b^±0.04
Hexyl acetate	4.27^a^±0.14	9.74^b^±1.70	9.50^a^±1.46	36.99^b^ ±2.38	1.85^a^±0.16	1.89^a^±0.17	1.06^a^±0.20	2.40^b^±0.36
Octyl acetate	1.02^a^±0.18	1.09^a^±0.23	0.25^a^ ±0.04	0.60^b^±0.09	0.27^b^±0.04	0.17^a^±0.01	0.24^a^±0.04	0.24^a^±0.01
**Aromatic esters (μg/L)**								
Ethyl benzoate	0.33^b^ ±0.01*^1^	0.27^a^±0.04*^1^	0.17^a^ ±0.01	0.18^a^±0.02	0.80^a^±0.05	0.72^a^±0.08	0.85^a^±0.08	0.69^a^±0.06
Ethyl phenylacetate	4.91^b^ ±0.39	2.73^a^ ±0.23	3.02^b^ ±0.13	1.92^a^±0.14	5.91^a^±0.71	5.47^a^±0.83	10.45^a^±0.49	10.74^a^±1.46
Ethyl 3-phenylpropanoate	0.87^b^ ±0.07	0.51^a^±0.09	0.32^b^ ±0.05	0.15^a^±0.03	0.41^a^±0.12	0.32^a^±0.06	0.30^a^±0.03	0.24^a^±0.03
Ethyl salicylate	<0.10	<0.10	<0.10	<0.10	0.31^a^±0.29	0.31^a^±0.06	0.28^a^±0.02	0.28^a^±0.03
Hexyl phenylacetate	0.41^a^ ±0.06*^1^	0.66 ^b^ ±0.08*^1^	0.44^a^±0.10	0.55^b^±0.03	0.67^a^±0.02	0.74^b^±0.02	0.36^a^±0.03	0.38^a^±0.03
**Minor ethyl esters of odd carbon number acids (μg/L)**							
Ethyl valeriate	0.51^b^±0.03	0.38^a^±0.02	0.75^a^±0.04	0.67^a^±0.05	1.07^b^±0.03	0.85^a^±0.07	1.57^a^±0.12	1.53^a^±0.08
Ethyl hepatanoate	0.13^a^±0.01*^1^	0.34^b^±0.09*^1^	<0.05	0.13 ±0.00	0.45^a^±0.08*^1^	0.39^a^±0.08*^1^	0.77^a^±0.06	0.71^a^±0.10
**Minor isamyl ester (μg/L)**								
Isoamyl butyrate	0.51^b^±0.03	0.35^a^±0.06	0.28^b^±0.01*^1^	,0.22^a^±0.02*^1^	0.56^a^±0.04*^1^	0.55^a^±0.03*^1^	0.22^a^±0.01 *^1^	0.51^b^±0.04 *^1^
Isoamyl isovalerate	<0.05	<0.05	<0.05	<0.05	<0.05	<0.05	<0.05	<0.05
Isoamyl hexanaote	<0.05	<0.05	<0.05	<0.05	<0.05	<0.05	<0.05	<0.05
Isoamyl octanoate	0.50^a^ ±0.07	6.13^b^±0.97	1.04^a^±0.15	2.44^b^±0.12	0.56^b^±0.12	0.31^a^±0.08	0.54^a^±0.08	0.45^a^±0.05
**Methyl esters (μg/L)**								
Methyl isovaleriate	<0.05	<0.05	<0.05	<0.05	<0.05	<0.05	<0.05	<0.05
Methyl hexanaote	0.53^a^±0.08	0.56^a^±0.08	0.46^a^±0.05	0.63^b^±0.04	0.48^a^±0.08	0.37^a^±0.06	0.54^a^±0.06	0.84^a^±0.15
Methyl octanoate	0.12^a^±0.01*^1^	0.25^b^±0.03*^1^	0.12^a^ ±0.01*^1^	0.15^b^±0.01*^1^	0.14^a^±0.02	0.12^a^±0.02	0.14^a^±0.01 *^1^	0.15^b^±0.01 *^1^
Methyl decanoate	0.13^a^±0.00*^1^	0.50^b^±0.11*^1^	0.13^a^ ±0.01*^1^	0.15^a^ ±0.01*^1^	0.11^a^±0.01	0.11^a^±0.01	0.12^a^±0.01	0.10^a^±0.00
Methyl laurate	<0.21	<0.21	<0.21	<0.21	<0.21	<0.21	<0.21	<0.21
Methyl myristate	<0.24	<0.24	<0.24	<0.24	<0.24	<0.24	<0.24	<0.24
**Minor esters of higher alcohols and other aliphatic acids than acetic acids (μg/L)**								
Propyl propionate	0.69^b^±0.16	0.36^a^±0.04	nd	nd	0.64^a^±0.05	0.57^a^±0.12	0.25^a^±0.03	0.28^a^±0.06
Isobutyl propionate	20.11^a^±1.16	23.25^b^±1.28	31.95^a^±1.80	32.60^a^±2.39	33.12^a^±2.68	32.11^a^±1.06	31.31^a^±1.62	36.53^b^±3.08
Isobutyl butyrate	<13.00	<13.00	<13.00	<13.00	<13.00	<13.00	<13.00	0.11 ±0.01
Pentyl butyrate	1.08^a^±0.23	1.50^a^±0.31	0.95^a^±0.27	0.91^a^±0.23	1.31^a^±0.09	1.31^a^±0.16	0.92^a^±0.11	1.04^a^±0.13
Isobutyl hexanoate	0.79^b^ ±0.01	0.73^a^±0.04	0.60^a^±0.02	0.61^a^±0.02	0.67^a^±0.02	0.65^a^±0.05	0.63^a^±0.03	0.63^a^±0.03
**Miscellaneous minor esters (μg/L)**								
Methyl-trans-geranoat	0.21^b^±0.02*^1^	0.18^a^±0.01*^1^	0.17^a^±0.01	0.19^a^±0.02	0.17^a^±0.01	0.17^a^±0.02	0.18^a^±0.02	0.20^a^±0.01
Ethyl (E) 2-decenoate	0.13^a^±0.02	0.31^b^±0.03	0.17^a^±0.03	0.24^a^±0.05	0.16^a^±0.01	0.16^a^±0.02	0.12^a^±0.02	0.13^a^±0.02

All the parameters are given with their mean and standard deviation. For each volatile substance, different letters in the same variant (grape and origin) indicate significant differences between variants. (ANOVA p≤0.05; *^1^ Mann Withney U-Test p≤0.100); not detectable.

Our data reveal considerable differences in the important acetate esters. The spontaneously fermented wines Grüner Veltliner B, Grüner Veltliner K, and Pinot noir wines showed on average significantly higher concentrations of isoamyl acetate, while in Zweigelt the concentrations were significantly lower. The results of hexyl acetate showed significantly higher levels for the spontaneously fermented white wines and Pinot noir than the inoculated wines except Zweigelt, where the difference was not significant. The content of isobutyl acetate was significantly higher in all spontaneously fermented variants than in inoculated wines.

There were significant differences among the ethyl esters in Grüner Veltliner, with consistently higher ethyl ester contents in the spontaneously fermented wines than the inoculated wines, except for ethyl octanoate (not significant) and ethyl butyrate (significant) having higher content in the inoculated Grüner Veltliner B. Diethyl succinate was consistently higher in the inoculated wines (significant with Grüner Veltliner B, Grüner Veltliner K and Pinot noir; Table 3).

In the case of the aromatic esters, significant differences were found only in the Grüner Veltliner wines. While ethyl phenylacetate and ethyl 3-phenylpropanoate were produced more in the inoculated wines, the concentrations of hexyl phenylacetate were higher on average in the spontaneously fermented wines.

In the group of branched aliphatic esters, ethyl isovalerate content was higher in the inoculated wines which resulted in significant differences in the total content of this chemical group for white wines. For the ethyl esters of odd-carbon-number acids, the inoculated wines (except for Pinot noir) of ethyl valerate showed higher average concentrations (significant in Grüner Veltliner B). The results of ethyl heptanoate, however, were more heterogeneous. In the case of the methyl esters, there were significant differences only in Grüner Veltliner B and K. In the group of quantitatively less important isoamyl esters, the concentrations in isoamyl octanoate were 10 times higher in the spontaneously fermented wines of Grüner Veltliner B than in inoculated wines, but the inoculated variants of the red wines showed higher concentrations than the respective spontaneously fermented wines. The group of minor esters of higher alcohols and other aliphatic acids (except acetic acids) and miscellaneous minor esters showed small differences in concentration between the spontaneously fermented and the inoculated variants. In contrast, significant differences were observed between the Grüner Veltliner B and Pinot noir, with higher concentrations of propyl propionate, isobutyl hexanoate and methyl-trans-geranote in the inoculated wines of Grüner Veltliner B, while the spontaneously fermented wines of Grüner Veltliner B and Pinot noir showed higher concentrations in isobutyl propionate (Table 3).

### 3.8 Diversity in aroma

Table 4 shows the sum concentrations of the different aroma groups for white, red and total wine and the differences in concentrations between white and red wine. The sum of acetates of higher alcohols was significantly higher in spontaneously fermented than in inoculated white wines, red wines and average content of red and white wines together. Significant differences in the sum of minor isoamyl ester, methyl esters and miscellaneous minor esters were found for the white wine variants but not for the red wine and total wine variants. It is noteworthy that the difference in aroma concentration between the red wine variants and white wine variants was always higher in the spontaneous fermentation variants, except for the esters of branched aliphatic acids and minor ethyl esters of odd carbon number acids.

**Table 4:** Sum concentration for white. red. total wine variants and differences between white and red variants of the different aroma groups.

Variante	WWY	WWSP	RWY	RWSP	TW Y	TW SP	Difference between WW Y and RWY	Difference between WW SP and RW SP
Minor acids (mg/L)	9.32^a^	13.17^a^	5.52^a^	5.24^a^	7.20^a^	9.36^a^	-3.8	**-7.93**
Higher alcohols (mg/L)	189.49^a^	173.07^a^	326.21^a^	358.59^a^	238.33^a^	243.89^a^	136.72	**185.52**
Major ethyl esters (mg/L)	220.78^a^	144.35^a^	218.35^a^	252.61^a^	214.87^a^	183.97^a^	-2.43	**108.26**
Minor ethyl esters (mg/L)	1309.02^a^	1502.18^a^	344.62^a^	374.02^a^	856.43^a^	947.35^a^	-964.4	**-1128.16**
Esters of branched aliphatic acids (μg/L)	30.68^b^	11.48^a^	10.84^a^	9.02^a^	20.39^b^	9.72^a^	**-19.84**	-2.46
Higher alcohol acetate (μg/L)	229.07^a^	525.23^b^	155.55^a^	282.30^b^	182.26^a^	401.97^b^	-73.52	**-242.93**
Aromatic esters (μg/L)	5.33^b^	3.58^a^	10.17^a^	9.95^a^	7.13^a^	6.11^a^	4.84	**6.37**
Minor ethyl esters of odd carbon number acids (μg/L)	0.66^a^	0.72^a^	1.93^a^	1.74^a^	1.16^a^	1.11^a^	**1.27**	1.02
Minor isoamyl ester (μg/L)	1.25^a^*^1^	4.65^b^*^1^	1.02^a^	1.00^a^	1.08^a^	2.90^a^	-0.23	**-3.65**
Methyl esters (μg/L)	1.22^a^	1.59^b^	1.24^a^	1.32^a^	1.14_a_*^1^	1.38^b*1^	0.02	**-0.27**
Minor esters of higher alcohols and other aliphatic acids than acetic acids (μg/L)	41.08^a^	42.98^a^	47.43^a^	49.56^a^	41.30^a^	43.08^a^	6.35	**6.58**
Miscellaneous minor esters (μg/L)	0.17^a^	0.23^b^	0.16^a^	0.17^a^	0.16^a^	0.19^a^	-0.01	**-0.06**
Sum Esters (without major ethyl esters) (μg/L)	1618.48^a^	2092.66^a^	572.97^a^	729.06^a^	1111.04^a^	1413.98^a^	-1045.51	**-1363.6**

Parameters are given with their mean. For each volatile substance different letters in the same variant (WW = white wine; RW = red wine; TW = total wine) indicate significant differences between variants (ANOVA p≤0.05; *^1^ Mann Withney U-Test p≤0.05), bold numbers indicate bigger differences between red and white wine fermentations.

### 3.9 Statistical evaluation of chemical analyses

**Fig 4:**
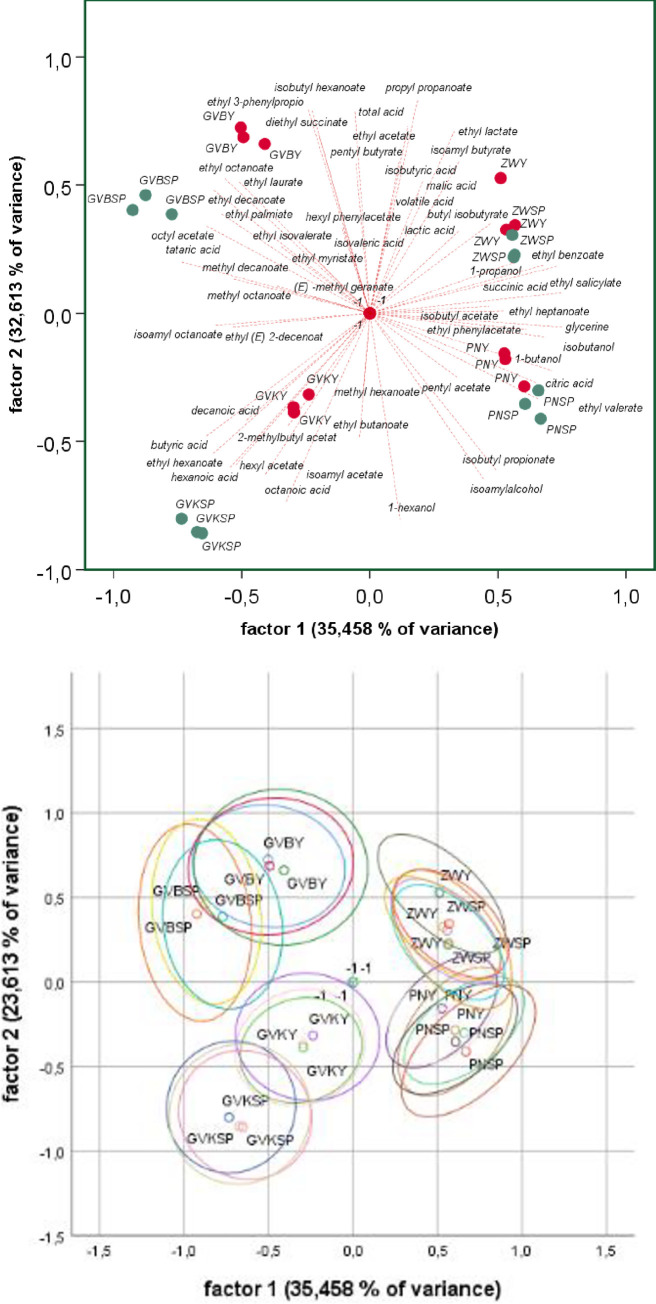
Chemical footprint all variants. (A) biplot (B) with 95% confidence ellipse: GV B = Grüner Veltliner Burgenland, GV K = Grüner Veltliner Klosterneuburg, ZW = Zweigelt, PN = Pinot noir; red dots: inoculated variants; green dots: spontaneously fermented variants.

Principal Component Analysis (PCA) was used to investigate the correlation between grape origin, varietal, the chemical composition of the wines and the fermentation modality. The analysis was performed on all quantified chemical parameters (Fig 4). The first two principal components (PC1 and PC2) explained 68,07% of the variance in the data set. PC1, which explained 35,46% of the variance, allowed for the separation of the red and white cultivars, while PC2 (32,61%) was able to distinguish the wines mainly according to the origin, resulting in a clear separation of the Grüner Veltliner B and Zweigelt from Burgenland from the Grüner Veltliner K and Pinot noir from Lower Austria.

**Fig 5:**
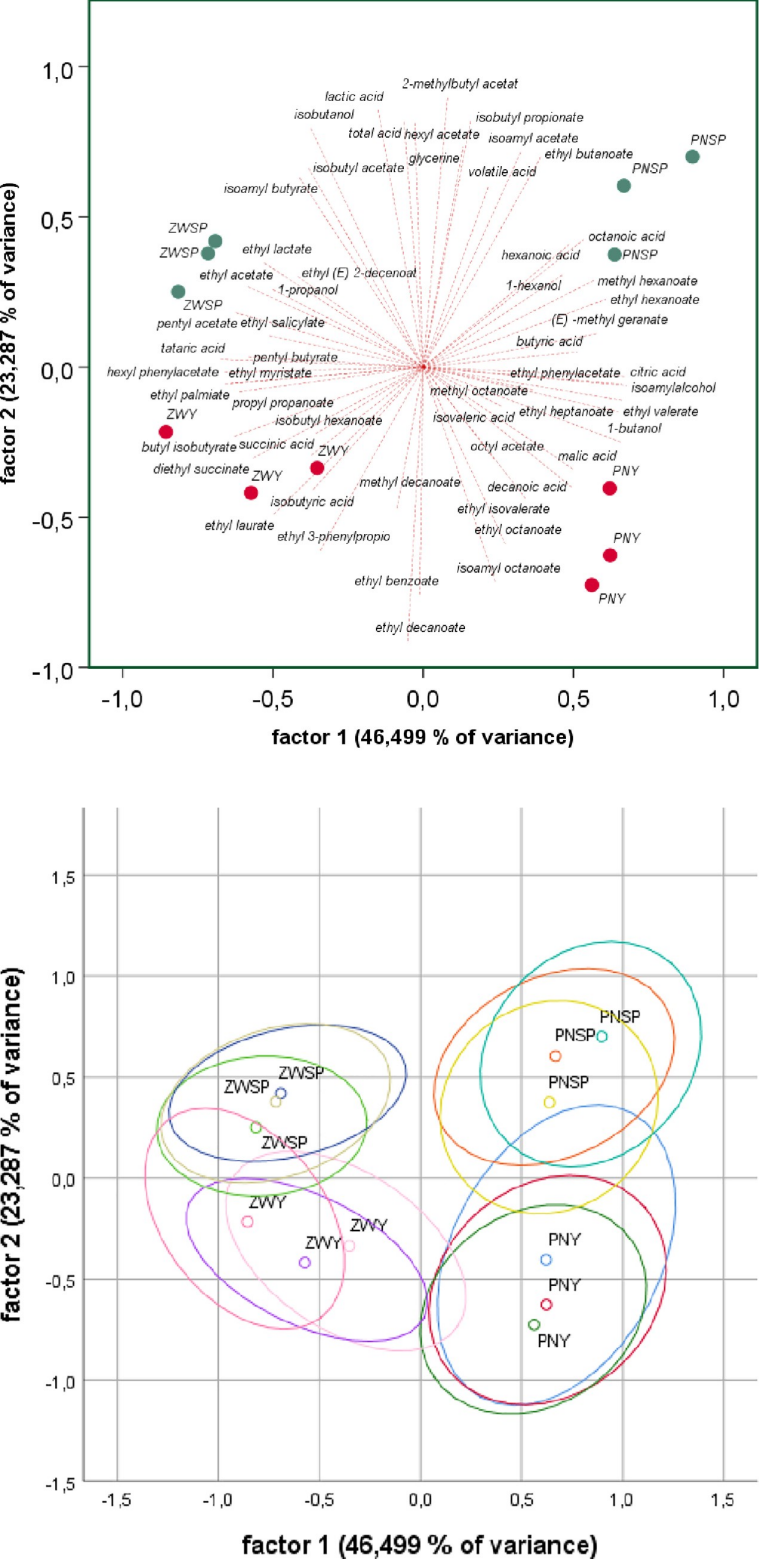
Chemical footprint red variants. (A) biplot (B) with 95% confidence ellipse: ZW = Zweigelt, PN = Pinot noir; red dots: inoculated variants; green dots: spontaneously fermented variants.

**Fig 6:**
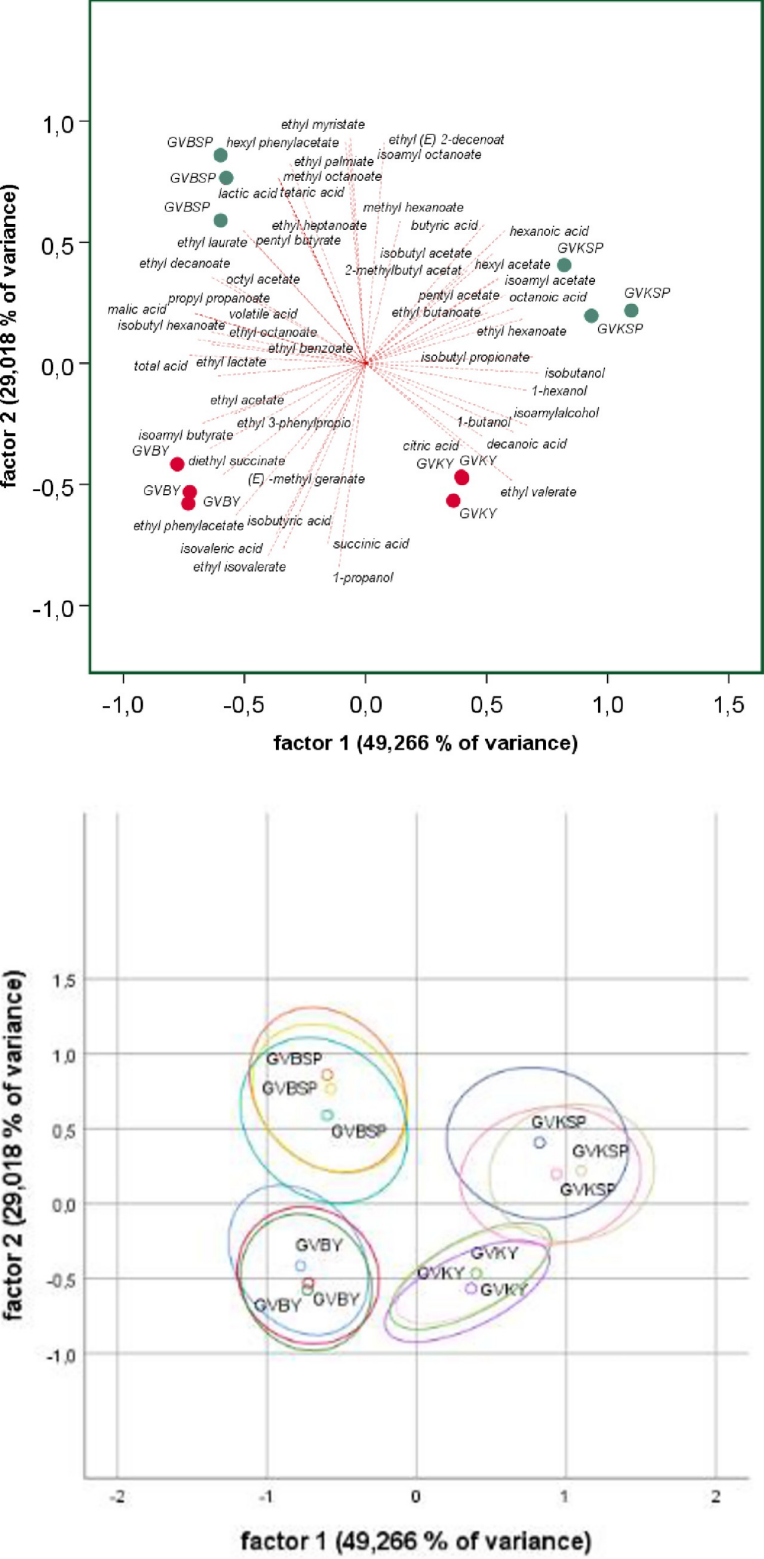
Chemical footprint white variants. (A) biplot (B) with 95% confidence ellipse: GV B = Grüner Veltliner Burgenland, GV K = Grüner Veltliner Klosterneuburg; red dots: inoculated variants; green dots: spontaneously fermented variants.

For further analysis, the red (Fig 5) and white wine (Fig 6) data sets were visualized independently. Regarding the two red wines, PCA showed that grape varietals had a strong effect on the chemical composition. Pinot noir and Zweigelt were separated along PC1, which explained 46,49% of the variance, while PC2 (23,28%) was able to distinguish the wines produced by spontaneous fermentation from the inoculated wines. There was consistent overlap in the confidence intervals between the fermentation styles while no overlap was observed for the grape cultivars. Concerning the loadings plot, total acid, lactic acid, volatile acid, glycerine, isobutanol, and some esters like hexyl acetate, 2-methylbutyl acetate, isobutyl propionate, isoamyl acetate, ethyl butanoate clustered on the side of the spontaneously fermented wines while mainly minor esters like methyl decanoate, ethyl 3-phenylpropanoate, ethyl benzoate, ethyl decanoate clustered with the inoculated wines. Heterogeneous groups of chemicals were responsible for the separation according to the origin of wines.

For the white wines, the clustering indicated that origin and fermentation modality had a strong effect on the chemical composition. PC1 which explained 49,26% of the variance confirmed the separation of the Grüner Veltliner from Lower Austria and Burgenland (Fig 6), while similar to the red wines, the spontaneously fermented and the inoculated wines were separated along PC2, which explained (29,02%) of the variance. However, the confidence ellipse showed a slight overlap between the two fermentation styles but no overlap between the origin of wines. Succinic acid, 1-propanol, isovaleric acid, isobutyric acid, ethyl isovalerate clustered together with the inoculated wines while a bigger number of middle and long chain esters and some acids clustered onside of the spontaneously fermented wines.

## 4 Discussion

Much anecdotal evidence and a strong belief in the industry show that "non-inoculated fermentations" exhibit a larger chemical space than inoculated fermentations. This anecdotal evidence and these widely held beliefs are highly contestable, as they have never been properly investigated, and are thus without scientific evidence. Therefore, our study aimed to evaluate whether inoculated fermentations of different juices from different regions really end up with a narrower, less diverse chemical profile than those that are spontaneously fermented. Our data demonstrate that both the spontaneous and inoculated fermentations were largely dominated by the same species (OTU-852 bp) towards the end of fermentation. Based on the evolution of this OTU, well-established trends in wine fermentation [33–35] as well as published ITS1-5.8S rRNA-ITS2 gene sizes [36, 37], we can infer that this OTU represents *S*. *cerevisiae* or its natural hybrids such as *S*. *cerevisiae var*. *bayanus*. Indeed, several studies relying on culture-dependent approaches as well as high throughput sequencing [38] confirm that as wine fermentation (spontaneous or inoculated) progressed, the fungal community diversification between samples narrowed, mainly due to the dominance of *S*. *cerevisiae*. The rate of establishment of this OTU, as well as the evolution of the fungal community, differed between the ferments, with a notable influence of these dynamics on the chemical signature of the final wines. This is further strengthened by the fact that the inoculated juices consistently showed more dominance by this OTU, also confirming that the inoculation had the desired effect of more rapid implantation of a strong fermentative yeast.

The differences in the chemical profiles cannot be attributed to certain species/strains, such differences can rather be attributed to the fungal community. Due to the high number of treatments, it was difficult to identify the yeast species present in grape must. However, population dynamics of fungal community was monitored throughout the fermentation (Fig 2). As it has been displayed in results, in all inoculated treatments the OTU-852 bp established well and dominated the middle and end of fermentation while in some spontaneous treatments such as ZW-B-SP, this OTU accounted for less than 30% of the population at end of fermentation. According to these results and the previous work done in our group [39], the OTU of 852 bp is most likely *S*. *cerevisiae*. These results, confirms the point that the background microbiota will not only affect the establishment of *S*. *cerevisiae*, but also will influence the chemical profiles of wines.

When focusing on the samples that fermented to dryness, the GV-K and the Zweigelt wines showed generally similar population dynamics during fermentation in the spontaneous and inoculated ferments while PN samples showed clear differences in the fungal community structure between the spontaneous and inoculated fermentation from the start. This difference in fermentation species dynamics correlated with the diversity of chemical profiles: Similar population dynamics led to chemically more similar wines. The data show that the implantation and dominance of OTU-852 bp in the GV-K and ZW was curtailed, suggesting a strong competition by the natural microbiota, in particular, OTUs 470 bp and 759 bp, which were in high abundance in both types of samples. Since both the spontaneous and inoculated GV-K and ZW fermented to dryness in a short period, we can deduce that the species represented by these latter mentioned OTUs are moderate to strong fermenters. Although the dominance of *S*. *cerevisiae* in both spontaneous and inoculated fermentations is generally deemed essential to ferment wine to dryness [40], some non-*Saccharomyces* species e.g. *Candida stellata* and *Kloeckera apiculata* (anamorph, *Hanseniaspora uvarum*) are not always suppressed by inoculated *S*. *cerevisiae* strains [16]. A comparison of the spontaneous and inoculated PN variants shows that in both cases OTU-852 bp established well and dominated both fermentations from the middle of fermentation although with less abundance in the spontaneous variants. Seemingly, the persistence of the other OTUs albeit at lower abundance allowed for sufficient contribution to the final aroma profile. Moreover, although the OUT-852 bp is dominant in both the inoculated and spontaneous variants, it is important to note that the strains present could be different. Indeed, studies have shown that in spontaneous fermentation multiple strains of *S*. *cerevisiae* may be present throughout fermentation [13, 41].

Regarding the overall effect of inoculation on chemical profiles of wines, our data show that the average content of some compounds such as acids (malic, succinic and isobutyric acid) and esters (diethyl succinate, ethyl isovalerate, ethyl 3-phenylpropionate and ethyl valeriate) was higher in all inoculated wines whereas the average content of alcohols (isobutanol, isobutyl acetate) and important spoilage indicators like volatile acid and ethyl acetate, was higher in all spontaneously fermented wines. These data suggest that it might be possible to consistently distinguish between spontaneous fermentations and fermentations inoculated with ADY. However, these results need to be supported by further studies. Remarkably, our data show that some of the most significant differences between the fermentation styles were observed in the content of minor esters. Significant differences were observed in the sum content of esters of branched aliphatic acids (higher in inoculated white and total wines), aromatic esters (higher in inoculated white wines), minor isoamyl esters (higher in spontaneous fermented white wines), methyl esters (higher in spontaneous fermented white and total wines) and miscellaneous minor esters (significant higher for spontaneous fermented white wines). This suggests that these groups of compounds should be considered in future studies on the influence of fermentation dynamics on wine composition. It should also be noted that some of the reported ester compounds have well-known contributions to wine aroma which underlines the importance of quantifying these compounds [42].

When comparing the two Grüner Veltliner wines, it is evident that despite being inoculated with the same ADY, the two wines produced are chemically different, suggesting that regional delineation is not always diminished by the use of the same starter culture. This could also be attributed to the major differences in the indigenous fungal diversity and population dynamics. ARISA does not allow for the identification of species and strains, however, the fungal community profiles of the two ferments were well separated on a PCA bi-plot (Fig 1) showing convergence only at the end of fermentation. These data align with phylogenetic surveys that have been performed with high-throughput amplicon sequencing technologies on spontaneous wine fermentations [38, 43]. In addition to the fungal community structure, it is important to acknowledge the possible contributions of other factors, such as differences in the initial juice matrix, agricultural practice and interspecific interactions, towards regional flavours and aromas of wine. Indeed, the basic chemical parameters of the juices, show that the key factors (e.g. pH, total acidity and YAN) that can influence yeast growth, interactions, population dynamics as well as wine flavour and aroma were different in the two Grüner Veltliner juices.

Looking into more detail and considering the differences in the concentrations of volatile compounds reveals that the difference between spontaneously fermented red and white wines is more pronounced than for the inoculated variants (Table 3). This holds for all aroma groups except the esters of branched aliphatic and the minor ethyl esters of acids with odd carbon numbers. Thus, our data suggest that inoculation with ADY, even in the extreme case (red vs. white), limits the aromatic fingerprint of the wines compared to spontaneous fermentation. This assumption is further supported by Figs 4–6. In Fig 4, the inoculated variants fall within a limited range of -0.5 to 0.5 to a factor of 1, while the spontaneous variants scatter in the range of -0.9 to 0.6. Figs 5 and 6 show that this limitation in the aroma diversity of the wines also applies when white wines and red wines are compared with each other. It has to be noted, that the fermentation of the GV B SP variant got stuck during fermentation. Still, this does not invalidate the observations described for the other variants. To our knowledge, this is the first study evaluating the question of flavor variability concerning spontaneous versus inoculated fermentations. However, it is important to note that our data do not support any direct correlation between chemical profiles and microbial analysis.

Lastly, the samples in the chemical fingerprint clustered according to the variety, origin and fermentation style (Fig 4), thus, a clear link between the origin of samples and microbial fingerprint is evident. This result is in agreement with previous studies which confirmed that grape and wine microbiota exhibit regional patterns, correlating with wine chemical composition and that aromatic profile of wine is strongly affected by grape microbial composition [36, 44, 45].

## 5 Conclusion

In this study, we highlight the chemical distinction between spontaneously fermented and inoculated wines from different origins and varieties in Austria. Our data demonstrate that the use of ADY is a limiting factor for chemical flavour diversity. However, reduced diversity in chemical profiles does not automatically suggest reduced sensory diversity. The number of flavour active compounds in wines is high, and the non-linear perceptual interactions between these compounds suggest that sensory spaces may be more dependent on relative concentrations than on the diversity of chemical fingerprints. It also remains to be evaluated how the use of different wine yeast strains would impact the observed reduction in chemical diversity, which will require further studies including sensory analysis. The reduced chemical diversity is likely due to the rapid dominance of one yeast strain (probably *S*. *cerevisiae* or its natural hybrids such as *S*. *cerevisiae var*. *bayanus)*, as evidenced by the convergence of the fungal community profiles. Of course, more data are needed, and our study has clear weaknesses (including the lack of species-specific data), but that does not invalidate the approach or conclusion.

## Supporting information

S1 Fig Fermentation dynamic. (A) Fermentation dynamic GV B; (B) Fermentation dynamic GV K; (C) Fermentation dynamic PN; (D) Fermentation dynamic ZW.
(PDF)

S1 Table Calibration and validation sheet of aroma analysis. (PDF)

S1 Data (JPEG)
