# Supplementary material for: Inoculation of grape musts with single strains of Saccharomyces cerevisiae yeast reduces the diversity of chemical profiles of wines
Source: PLoS One. 2021 Jul 22;16(7):e0254919. doi: 10.1371/journal.pone.0254919 (PMC8297920; doi:10.1371/journal.pone.0254919)
Supplement: S1 Fig — (A) Fermentation dynamic GV B; (B) Fermentation dynamic GV K; (C) Fermentation dynamic PN; (D) Fermentation dynamic ZW. (PDF) [file pone.0254919.s001.pdf]

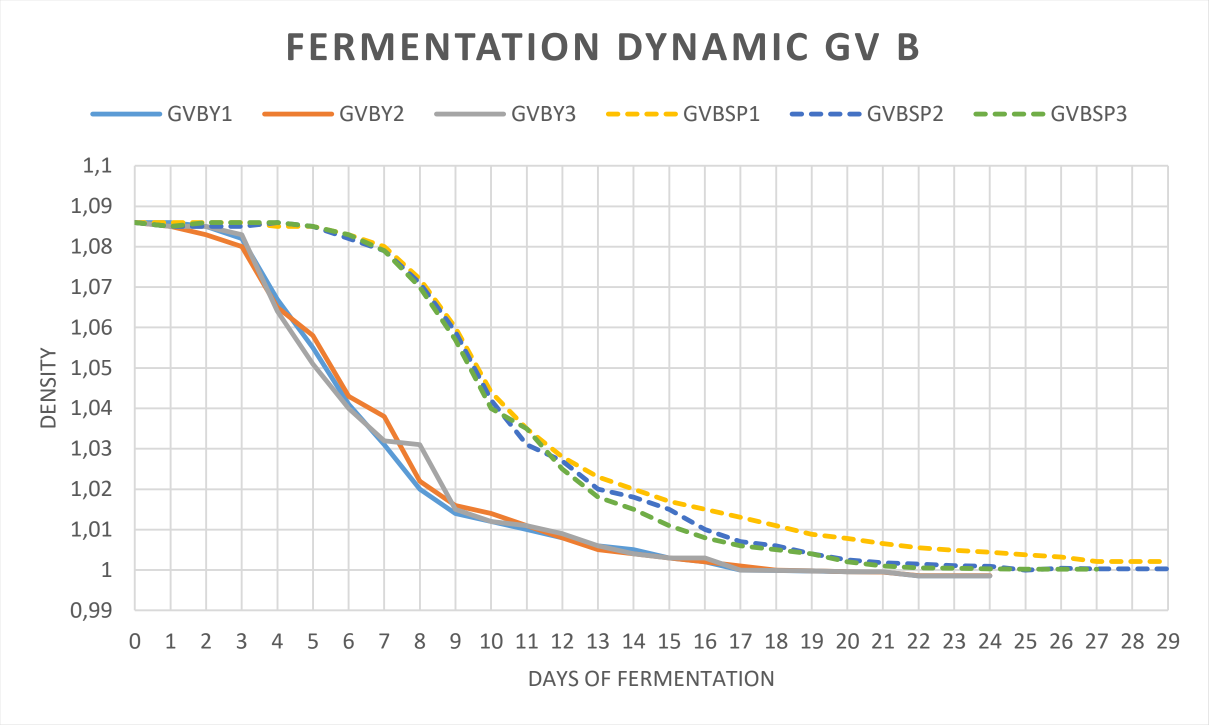

Figure A: Fermentation Dynamic GV B

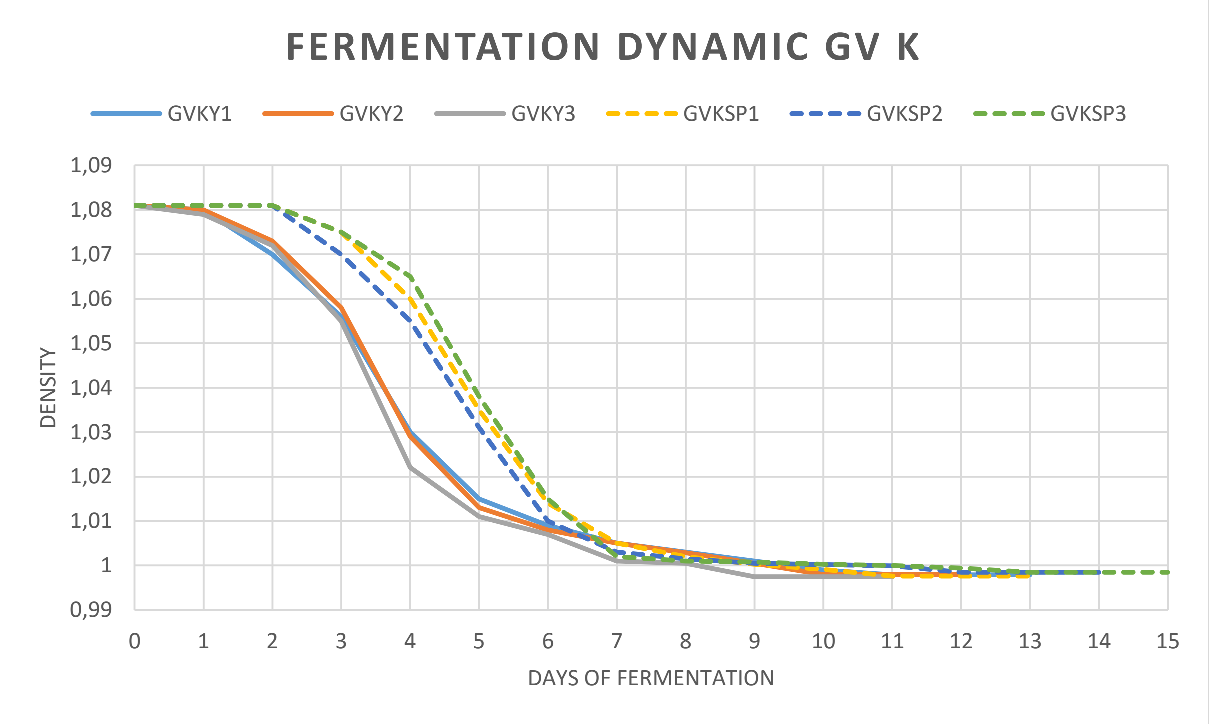

Figure B: Fermentation Dynamic GV K

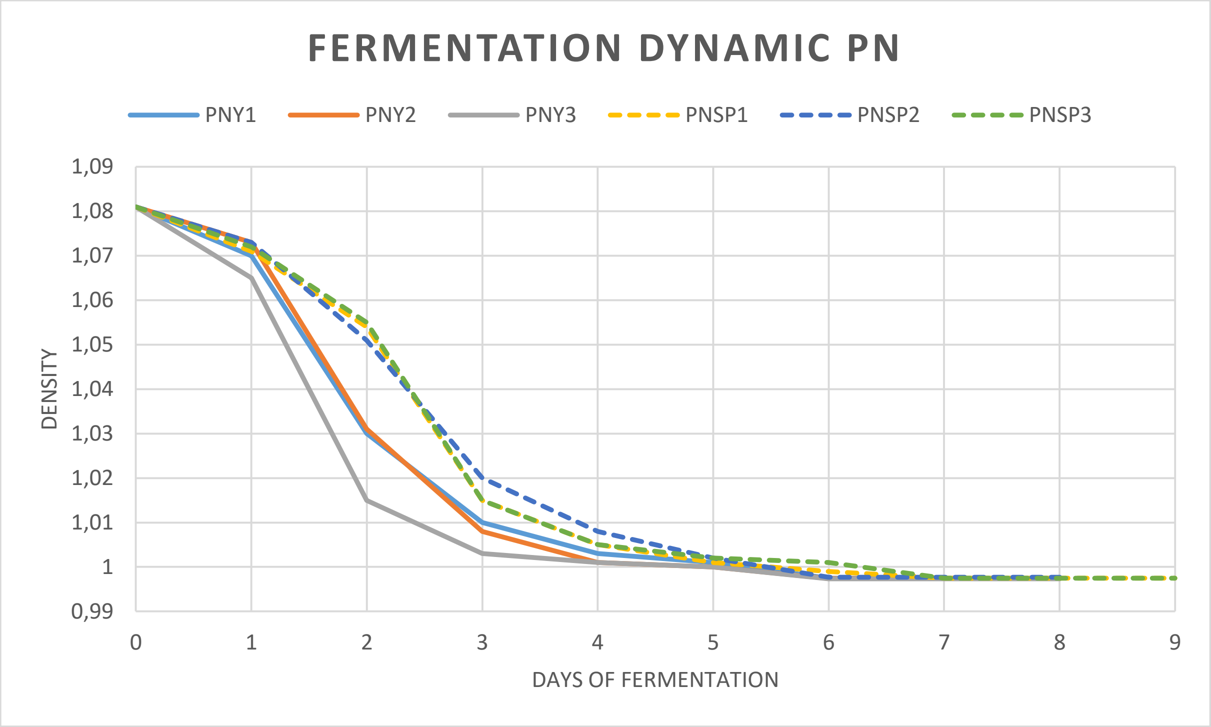

Figure C: Fermentation dynamic PN

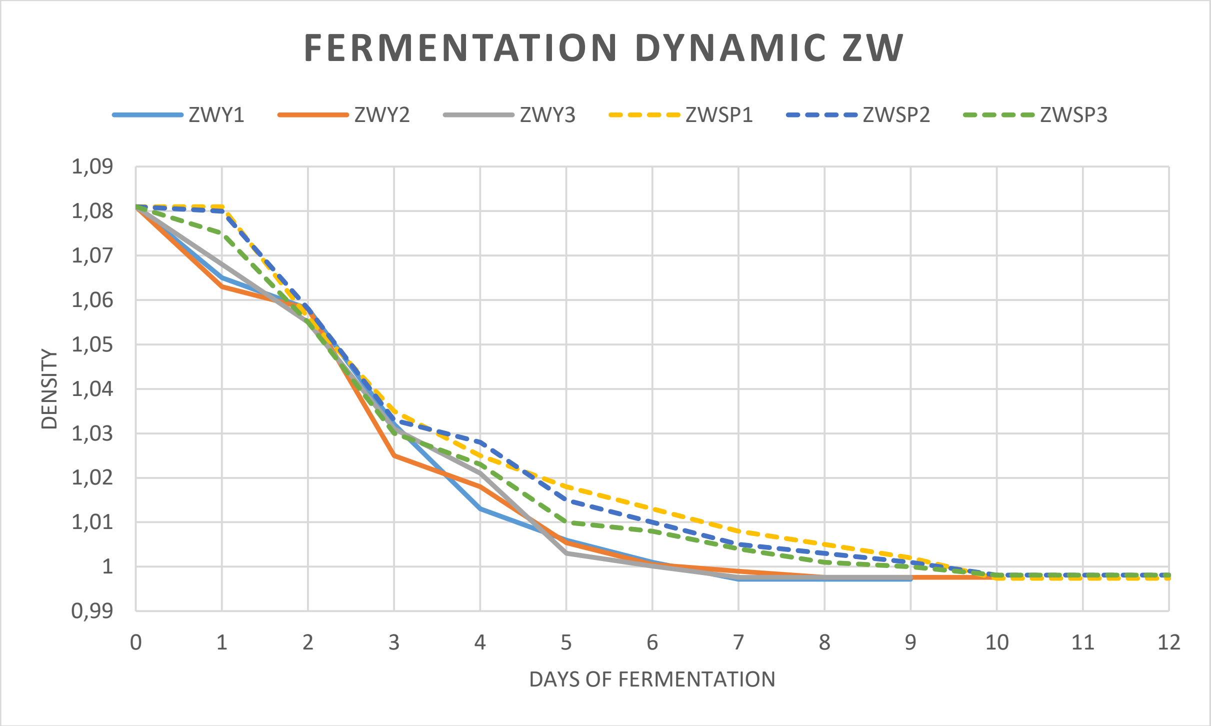

Figure D: Fermentation dynamic ZW
