# Supplementary material for: Inoculation of grape musts with single strains of Saccharomyces cerevisiae yeast reduces the diversity of chemical profiles of wines
Source: PLoS One. 2021 Jul 22;16(7):e0254919. doi: 10.1371/journal.pone.0254919 (PMC8297920; doi:10.1371/journal.pone.0254919)
Supplement: S1 Table — (PDF) [file pone.0254919.s002.pdf]

Supplementary Table 1: Calibration and validation sheet of aroma analysis:

Volatile substance (µg/l)	**Methode**	**Quantifier**	**Qualifier**	**Interner Standard**	**calibration area**	**linearity**	**RT (min)**	**repeatability**	**LOQ**	**LOD**
butyric acid (mg/l)	MV	60	73	d7-butyric acid	0.93-18635.76	0.998	34.29	7.1 %	0.932	0.280
isobutyric acid (mg/l)	MV	43	73, 88	d12-hexanoic acid	0.10-3.11	0.999	32.00	10.9 %	0.104	0.031
isovaleric acid (mg/l)	MV	60	87	d13-hexanol	0.06-5.82	0.998	35.51	4.8 %	0.058	0.017
hexanoic acid (mg/l)	MV	60	73, 87	d12-hexanoic acid	0.10-1.03	0.999	42.12	7.0 %	0.103	0.031
octanoic acid (mg/l)	MV	115	73	d12-hexanoic acid	0.03-2.93	0.999	49.65	15.0 %	0.029	0.009
decanoic acid (mg/l)	MV	129	73	d12-hexanoic acid	0.12-23.52	0.998	56.02	17.9 %	0.118	0.035
1-propanol (mg/l)	MV	31	42	d10-butanol	0.09-496.01	0.999	8.77	8.2%	0.010	0.003
isobutanol (mg/l)	MV	42	31	d10-butanol	0.91-91.79	0.999	10.89	5.7%	0.918	0.275
isoamyl alcohol (mg/l)	MV	70	43	d10-butanol	10.42-521.24	0.999	16.16	7.8%	10.425	3.127
1-butanol (mg/l)	MV	56	41	d10-butanol	0.06-2.79	0.998	13.24	7.6%	0.063	0.019
1-hexanol (mg/l)	MV	69	55	d13-hexanol	0.01-6.48	0.998	22.83	2.8 %	0.013	0.004
ethyl acetate (mg/l)	MV	61	43	d5-ethyl octanoate	9.83-196.57	0.999	4.47	7.0 %	9.829	2.949
ethyl butanoate	MME	71	88, 43	d5-ethyl valerate	12.5-2500.0	0.998	12.79	3.4 %	12.50	3.75
ethyl hexanoate	MME	88	99, 43	d5-ethyl hexanoate	13.2-2641.0	0.999	26.50	3.1 %	13.21	3.96
ethyl octanoate	MME	88	101, 127	d5-ethyl octanoate	16.4-3280.0	0.999	41.51	2.6 %	16.40	4.92
ethyl decanoate	MME	88	101	d5-ethyl decanoate	16.9-3385.8	0.999	54.96	8.2 %	16.93	5.08
ethyl laurate	MME	88	101, 157	d5-ethyl decanoate	0.3-50.8	0.998	66.98	15.7 %	0.25	0.08
ethyl myristate	MME	TIC	TIC	d5-ethyl decanoate	0.3-49.9	0.998	78.03	11.9 %	0.25	0.07
ethyl palmiate	MME	88	101, 157	d5-ethyl decanoate	3.9-55.9	0.998	88.05	13.0 %	3.92	1.18
diethyl succinate (mg/l)	MV	101	129	d5-ethyl octanoate	0.1-9.9	0.997	35.94	11.3 %	0.099	0.030
ethyl lactate (mg/l)	MV	45	75	d5-ethyl octanoate	0.10-51.78	0.998	22.09	6.5 %	0.104	0.031
butyl isobutyrate	MME	43	71	d5-ethyl hexanoate	1.0-11.0	0.999	22.91	9.5 %	0.98	0.30
ethyl isovalerate	MME	88	85, 57	d5-ethyl valerate	0.3-49.8	0.998	15.97	3.3 %	0.25	0.07
propyl isovalerate	MME	85	103	d5-ethyl hexanoate	0.1-10.0	0.998	22.70	[*2]	0.05	0.02
isobutyl acetate	MME	43	56	d5-ethyl valerate	12.7-2540.0	0.998	11.39	8.3 %	12.70	3.81
2-methylbutyl acetate	MME	43	70	d5-ethyl valerate	13.4-2680.0	0.997	17.62	3.7 %	13.40	4.02
isoamyl acetate	MME	43	70	d5-ethyl valerate	1.0-11.6	0.996	17.53	4.1 %	0.98	0.29
pentyl acetate	MME	43	70	d5-ethyl valerate	1.0-9.7	0.991	19.04	[*2]	0.10[*1]	
hexyl acetate	MME	43	56, 61	d5-ethyl hexanoate	1.3-263.0	0.999	27.39	3.2 %	1.32	0.40
octyl acetate	MME	43	70, 84	d5-ethyl decanoate	0.1-11.0	0.998	42.90	11.6 %	0.10[*1]	
ethyl benzoate	MME	105	122	d5-ethyl hexanoate	0.1-14.3	0.999	39.38	6.3 %	0.07	0.02
ethyl phenylacetate	MME	91	65	d5-ethyl octanoate	0.1-13.0	0.996	44.50	9.5 %	0.07	0.02
Ethyl (3)-phenylpropionate	MME	104	91	d5-ethyl octanoate	0.1-10.1	0.991	51.63	[*2]	0.10[*1]	
ethyl salicylate	MME	120	92	d5-ethyl octanoate	0.1-9.9	0.995	46.17	[*2]	0.10[*1]	
hexylphenyl acetate	MME	91	43, 136	d5-ethyl decanoate	0.-9.6	0.991	69.12	[*2]	0.10[*1]	
ethyl valerate	MME	85	88, 57	d5-ethyl valerate	0.1-10.4	0.998	33.83	4.4 %	0.13	0.04
ethyl heptanoate	MME	88	113	d5-ethyl valerate	0.1-10.5	0.999	33.83	3.9 %	0.05	0.02
isoamyl butanoate	MME	71	70, 43	d5-ethyl hexanoate	0.1-9.6	0.999	30.74	7.2 %	0.05	0.01

isoamyl isovalerate	MME	85	70	d5-ethyl octanoate	0.1-9.2	0.998	34.35	[*2]	0.05	0.01
isoamyl hexanoate	MME	70	43, 99	d5-ethyl octanoate	0.1-10	0.997	45.15	5.9 %	0.05	0.02
isoamyl octanoate	MME	70	127	d5-ethyl octanoate	0.1-10.1	0.997	58.06	13.8 %	0.05	0.02
methyl isovalerate	MME	74	85, 57	d5-ethyl valerate	0.1-10.4	0.999	11.54	[*2]	0.05	0.02
methyl hexanoate	MME	74	87, 43	d5-ethyl valerate	0.1-10.6	0.999	20.78	3.9 %	0.05	0.02
methyl octanoate	MME	74	87, 127	d5-ethyl hexanoate	0.1-8.7	0.999	35.80	6.6 %	0.04	0.01
methyl decanoate	MME	74	87, 143	d5-ethyl octanoate	0.1-10.6	0.999	50.04	5.9 %	0.05	0.02
methyl laurate	MME	74	87, 143	d5-ethyl decanoate	0.2-41.9	0.998	62.82	[*2]	0.21	0.06
methyl myristate	MME	74	87, 143	d5-ethyl decanoate	0.2-48.6	0.998	74.33	[*2]	0.24	0.07
Propyl propionate	MME	57	57, 75	d5-ethyl valerate	0.1-10.1	0.995	13.14	[*2]	0.10[*1]	
isobutyl propionate	MME	57	29	d5-ethyl valerate	0.3-52.1	0.998	16.85	8.3 %	0.26	0.08
isobutyl butyrate	MME	71	88	d5-ethyl hexanoate	13.0-2600.0	0.999	26.12	0.9 %	13.00	3.90
pentyl butyrate	MME	43	71, 70	d5-ethyl hexanoate	0.1-10.8	0.999	33.65	12.1 %	0.05	0.02
isobutyl hexanoate	MME	145	127, 61	d5-ethyl octanoate	0.1-10.4	0.997	47.87	4.8 %	0.05	0.02
Methyl (E)-geranoate	MME	43	69, 41	d5-ethyl hexanoate	0.1-9.8	0.997	41.95	[*2]	0.10[*1]	
Ethyl (E)-2-decenoate	MME	152	88	d5-ethyl octanoate	0.1-9.6	0.995	57.61	[*2]	0.10[*1]	

*1 has not be detected, was estimated

*2 could not be collected because it was not detectable in the validation wine
