# Supplementary figures and images for: Inoculation of grape musts with single strains of Saccharomyces cerevisiae yeast reduces the diversity of chemical profiles of wines
Source: PLoS One. 2021 Jul 22;16(7):e0254919. doi: 10.1371/journal.pone.0254919 (PMC8297920; doi:10.1371/journal.pone.0254919)

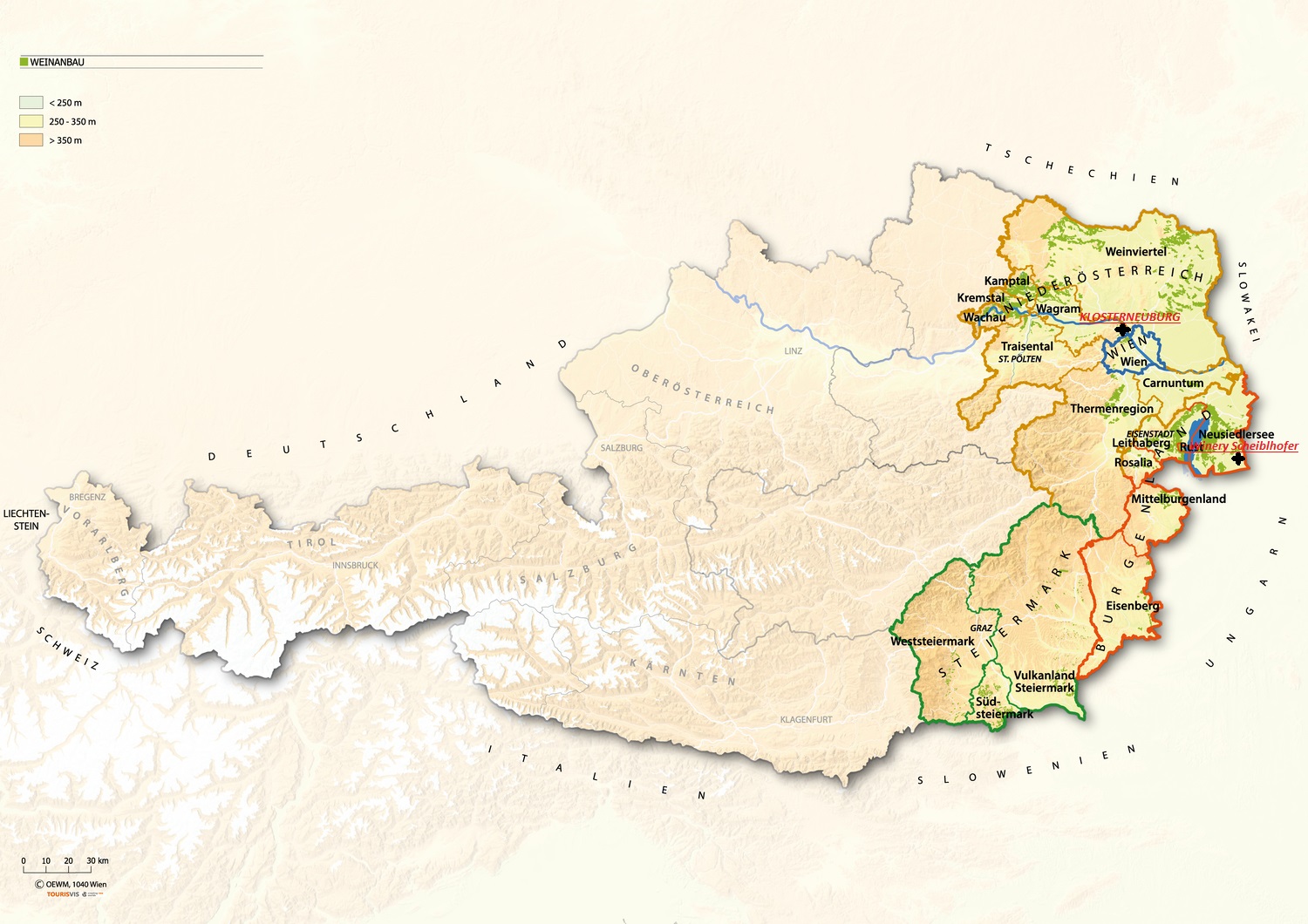

Supplement: S1 Data — (JPEG) [file pone.0254919.s003.jpeg]
